# Prognostic Value of FDG-PET/CT Parameters in Patients with Relapse/Refractory Multiple Myeloma before Anti-CD38 Based Therapy

**DOI:** 10.3390/cancers13174323

**Published:** 2021-08-27

**Authors:** Guillemette Fouquet, Myriam Wartski, Amina Dechmi, Lise Willems, Bénédicte Deau-Fischer, Patricia Franchi, Justine Descroocq, Paul Deschamps, Estelle Blanc-Autran, Jérôme Clerc, Didier Bouscary, Sylvain Barreau, Nicolas Chapuis, Marguerite Vignon, Anne-Ségolène Cottereau

**Affiliations:** 1 Assistance Publique-Hôpitaux de Paris Centre, Université de Paris, Service d’Hématologie Clinique, Hôpital Cochin, 75014 Paris, France; guillemette.fouquet@aphp.fr (G.F.); lise.willems@aphp.fr (L.W.); benedicte.deau-fischer@aphp.fr (B.D.-F.); patricia.franchi@aphp.fr (P.F.); justine.descroocq@aphp.fr (J.D.); paul.deschamps@aphp.fr (P.D.); didier.bouscary@aphp.fr (D.B.); marguerite.vignon@aphp.fr (M.V.); 2 Assistance Publique-Hôpitaux de Paris Centre, Université de Paris, Service de Médecine Nucléaire, Hôpital Cochin, 75014 Paris, France; myriam.wartski@aphp.fr (M.W.); amina.dechmi@aphp.fr (A.D.); jerome.clerc@aphp.fr (J.C.); 3 Hôpital Marie Lannelongue, Service de Médecine Nucléaire, 92350 Le Plessis-Robinson, France; estelle.blanc-ext@aphp.fr; 4 Assistance Publique-Hôpitaux de Paris Centre, Université de Paris, Service D’hémato-Biologie, Hôpital Cochin, 75014 Paris, France; sylvain.barreau@aphp.fr (S.B.); nicolas.chapuis@aphp.fr (N.C.)

**Keywords:** myeloma, immunotherapy, anti-CD38, FDG-PET/CT, prognostic factor, focal bone lesion

## Abstract

**Simple Summary:**

Anti-CD38 monoclonal antibody has improved the prognosis of relapsed/refractory multiple myeloma (RRMM) but some patients still experience early relapse with a poor outcome. We have here evaluated the predictive value of FDG PET/CT parameters prior to receiving anti-CD38-based therapy in RRMM cases. In 38 consecutive patients, the median PFS was 12.5 months and the median OS was not reached. The presence of >3 focal lesions (FLs) on relapse PET (*n* = 19) and the initial ISS were associated with both an inferior PFS (*p* = 0.0036 and *p* = 0.0026) and OS (*p* = 0.025 and *p* = 0.0098). In multivariable analysis, the ISS and >3 FLs were independent prognostic factors for PFS (*p* = 0.010 and *p* = 0.025). A prognostic score combining the ISS (1,2, or 3 points) and >3 FL (1 point) individualized an ultra-risk group (scored 3–4) with a median PFS and OS of 3.1 months and 8.5 months respectively vs. not reached for patients scored 1–2. Combined with the ISS, the presence of >3 FLs on PET could improve the risk stratification of RRMM patients.

**Abstract:**

Although anti-CD38 monoclonal antibodies have improved the prognosis of relapsed/refractory multiple myeloma (RRMM), some patients still experience early relapses with poor outcomes. This present study evaluated the predictive value of FDG PET/CT parameters for RRMM prior to initiating anti-CD38 treatment. We included 38 consecutive RRMM patients who underwent a PET/CT scan treated at our institution at relapse. The median PFS was 12.5 months and the median OS was not reached. 42% of the patients had an initial ISS score of 1, 37% of 2, and 21% of 3. The presence of >3 focal lesions (FLs, *n* = 19) and the ISS score were associated with inferior PFS (*p* = 0.0036 and *p* = 0.0026) and OS (*p* = 0.025 and *p* = 0.0098). Patients with >3 FLs had a higher initial ISS score (*p* = 0.028). In multivariable analysis, the ISS score and >3 FLs were independent prognostic factors for PFS (*p* = 0.010 and *p* = 0.025 respectively), and combined they individualized a high-risk group with a median PFS and OS of 3.1 months and 8.5 months respectively vs. not reached for the other patients. The presence of >3 FLs on PET was predictive of survival outcomes in patients with RRMM treated using CD38 targeted therapy. Combined with the initial ISS, an ultra-high-risk RRMM population can thus be identified.

## 1. Introduction

Immunotherapy with a monoclonal anti-CD38 antibody has improved the prognosis of relapsed/refractory multiple myeloma (RRMM) [1]. Isatuximab or Daratumumab in association with dexamethasone and/or chemotherapy can induce profound and durable responses. However, some patients experience early relapse and their prognosis is severe [2]. In the last few years, the added value of 2-deoxy-2-[18F]fluoro-D-glucose positron emission tomography/computed tomography (FDG PET/CT) over standard imaging has been demonstrated in many phases of this disease including the initial diagnosis and staging [3,4,5,6,7], restaging at relapse [8,9,10] and monitoring of the therapy response. This modality has also now been incorporated into the International Myeloma Working Group (IMWG) recommendations [3,11]. FDG PET/CT has further proven to be a promising prognostic tool. Durie et al. reported that the presence of extramedullary hypermetabolic lesions on FDG PET/CT was a negative prognostic factor both at the start of treatment and at relapse, in a series of 66 patients with MM at different disease stages [7]. Furthermore, a persistent abnormal FDG uptake within three months after an autologous stem cell transplant was reported to be predictive of a poorer PFS [12]. Notably, however, there are no specific data concerning the prognostic value of positive FDG PET/CT in the era of novel therapeutic agents, especially in the context of anti-CD38 immunotherapy administered at relapse or in refractory MM patients, which is expected to be increasingly used. Our present single-center study investigated the predictive value of FDG PET/CT parameters in a cohort of RRMM patients before anti-CD38 antibody therapy and compared its added value to the classical biological prognostic markers.

## 2. Materials and Methods

### 2.1. Patient Population

Consecutive patients with RRMM, defined using IMWG criteria, who underwent a baseline FDG PET/CT scan before commencing anti-CD38 based immunotherapy at our center (Cochin Hospital, Paris) between June 2019 and December 2020 were included in this study. The following MM history was recorded for these cases: initial date of diagnosis, ISS score at diagnosis, line of treatment before anti-CD38 therapy, and the presence of high-risk cytogenetics defined according to the revised international staging system (R-ISS) for MM, i.e., presence of del(17p), t(4;14), or t(14;16) using FISH analysis of bone marrow samples s [13]. Disease evaluation before anti-CD38 therapy included biochemical evaluation (monoclonal spike, serum-free light chain, albumin, and B2-microglobulin) and cytological evaluations (presence of abnormal circulating plasma cells on blood smear or with immunophenotyping when available). A high free light chain ratio was defined as a ratio between involved and uninvolved free light chains >120 [14].

### 2.2. PET/CT Acquisition and Analysis

A whole-body FDG PET/CT scan (top of head to feet, arms alongside body) was performed before initiating anti-CD38 based treatment. All FDG PET/CT was acquired in accordance with the European Association of Nuclear Medicine PET guidelines for FDG studies [15]. All FDG PET/CT scans were reviewed by two experienced nuclear medicine physicians (M.W. and A.S.C.), in accordance with the Italian Myeloma Criteria for PET Use (IMPeTUs) [12], first blinded and then in consensus. Positive focal lesions in the bone (FLs) were defined as a focally increased FDG uptake greater than the physiologic bone marrow uptake, with or without any underlying osteolytic lesion. Extramedullary disease (EMD) was defined as FDG-avid soft tissue not contiguous to the bone. The number and site of FLs and EMD were reported. Semiquantitative parameters, such as liver maximum standardized uptake value (SUVmax) as a reference organ, the bone marrow (BM) SUVmax defined in the lumbar vertebrae L3 (excluding FLs in this region), and SUVmax of the hottest bone and extramedullary lesions were annotated. The degree of FDG uptake was visually quantified in the BM out of FLs according to the 5-point Deauville scale (DS), which was referred to as the BM score (BMS).

### 2.3. Flow Cytometry

Circulating tumor plasma cells in the peripheral blood (PB) were detected using flow cytometry. Briefly, PB samples were collected in tubes containing EDTA and processed using a bulk-lysis procedure (Versalyse, Beckman Coulter, Brea, CA, USA) within 24 h of sample collection. Labeling was performed using a single 8-colors antibody combination: CD45-KrOr, CD138-PC5.5, CD56-AA700, CD27-PC7, CD19-ECD, CD200-AA750 (Beckman Coulter), Kappa-APC, and Lambda FITC (BD Biosciences, Franklin Lakes, NJ, USA), and CD38-BV421 (Biolegend, San Diego, CA, USA). First, membrane staining was performed and this was followed after a PBS washing procedure by intracytoplasmic labeling (anti-Kappa and anti-Lambda) using a Fix & Perm Cell Permeabilization Kit (Beckman Coulter, Brea, CA, USA). After the final PBS wash, the final cell pellet was resuspended in 500 µL of PBS before analysis using the flow cytometer (Navios, Beckman Coulter, Brea, CA, USA). From 200,000 to 1 million leucocytes were acquired for each patient to ensure the quality of the analysis and to allow a limit of detection to be set at ≥20 tumor plasma cells (0.01% to 0.002%, according to the number of leucocytes). Data were analyzed using Kaluza 2.1 software (Beckman Coulter, Brea, CA, USA).

### 2.4. Statistical Analysis

Data were prospectively collected from June to December 2020. Progression-free survival (PFS) was defined as the time from the date of antiCD38 therapy initiation to that of myeloma progression or death as a result of any cause. Overall survival (OS) was defined as the time from the date of antiCD38 therapy initiation until death as a result of any cause. Survival functions were calculated using Kaplan Meier estimates and comparisons between categories were made with the log-rank test. Univariate and multivariable analyses were performed using Cox proportional hazard models. The multivariable analysis included all parameters found to be significantly associated with PFS or OS in the univariate analysis. Characteristics of different populations were compared using Fisher’s exact test for discrete variables and the Mann-Whitney U test for continuous variables. For lesion SUVmax values, receiver-operating-characteristic (ROC) analysis was used to define the optimal cut-off for predicting the occurrence of an event (PFS or OS) by maximizing the Youden index.

### 2.5. Ethics Approval

This study is a non-interventional observational study and was approved by our institutional review board CLEP (n°: AAA-2021-08042), in accordance with the Helsinki declaration. In compliance with French legislation, every patient included in the analysis gave informed consent to the collection of clinical, biological, and radiological data as part of their ongoing medical care.

## 3. Results

### 3.1. Patient’s Characteristics

Thirty-eight patients, with a median age of 73 years, were included in the study cohort. Their characteristics are summarized in Table 1. The distribution of MM isotypes was as follows: IgG in 55% of cases, IgA in 21%, and light chain only in 24% (all kappa). An ISS initial stage of 1, 2 or 3 was found in 42% (*n* = 16), 37% (*n* = 14) and 21% (*n* = 8) of the patients, respectively. Almost 25% of the population (*n* = 8/33; 5 missing data) had a high-risk (HR) cytogenetic profile. The presence of circulating tumor cells in the plasma was analyzed by flow cytometry in 15 patients with five of these cases showing a positive result. The median time from the symptomatic MM diagnosis to the anti-CD38 treatment indication was 4.8 years (range 0.1–16.9 years). The median duration of the prior line of treatment before anti-CD38 immunotherapy was 3 (range 2–9 years) and 47% of the patients (*n* = 18) had undergone autologous stem cell transplantation. All patients received an anti-CD38 antibody associated with dexamethasone (isatuximab *n* = 6, daratumumab *n* = 32). An IMiD was administered in 50% of the patients and a proteasome inhibitor in 26% of the patients. With a median follow-up after commencing immunotherapy of 10 months (range, 1–21 months), 17 patients experimented a PFS event, among which 15 related to disease progression. The others patients are still under CD38 treatment. Nine patients died, 7 related to myeloma, 1 related to COVID-19, and 1 related to acute myeloid leukemia. The median PFS for the entire cohort was 12.6 months (95% CI 5.5–12.6) and the median OS was not reached.

### 3.2. PET/CT Characteristics and Correlation with Laboratory Findings

Prior to commencing anti-CD38 targeted therapy, 71% (*n* = 27) of the patients had visually detectable lesions on an FDG PET/CT scan, among whom 26 had at least one bone lesion (Table 2). Half of the patients (*n* = 19) had more than 3 FLs with a median SUVmax of 10.7 (IQR 5.3–18.6). EMD was present in 19% (*n* = 7) of all patients with a median SUVmax of 12.9 and was associated with bone focal lesions in all cases except for a single patient with EMD only. The extramedullary sites included pleuro-pulmonary (*n* = 2), muscle (*n* = 1), splenic (*n* = 1), liver (*n* = 2) and lymph nodes (*n* = 3).

We then wanted to compare the characteristics of patients according to the presence of ≤3 FLs or >3 FLs on PET/CT, as presented in Table 1. The presence of >3 FLs was associated with a higher serum β2microglobulin (B2M) level before starting anti-CD38 therapy (*p* = 0.015) and with a higher initial ISS score (*p* = 0.028). A trend was observed regarding differences in the free light chain concentration (dFLC), with a median of 206 mg/L in the group with >3 FLs but only 85 mg/L in the group with ≤3FLs (*p* = 0.08). The majority (83%) of the high FLC ratio was found in the >3 FLs group. We found that 75% of patients with HR cytogenetics (6/8) were in the >3 FLs group but this did not reach statistical significance (*p* = 0.24).

All 5 patients whose circulating tumor plasma cells were detected had at least one bone focal lesion detected on PET. Four of these cases had >3 FLs and the remaining patient had a single bone lesion associated with EMD. Among these 5 cases also, 4 had a PFS event (*p* = 0.089) and 3 had an OS event (*p* = 0.077).

### 3.3. Prognostic Factors 

The initial ISS remained a strong prognosticator for both PFS (*p* = 0.0026) and OS (*p* = 0.0098) whereas the presence of high-risk cytogenetics did not significantly correlate with these outcomes (Table 3).

**Figure 1:**
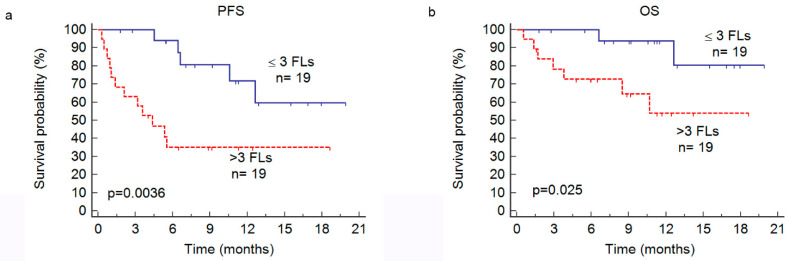
Kaplan–Meier estimates progression-free survival (PFS, **a**) and overall survival (OS, **b**) according to the presence of more than three focal lesions detected on PET (>3 FLs) or less/equal to three FLs (≤3 FLs).

At relapse, with regard to biological factors, only anemia correlated with both a poorer PFS (*p* = 0.030) and OS (*p* = 0.0096). The dFLC significantly impacted only the OS (*p* = 0.013). Concerning FDG PET/CT parameters, the presence of >3 FLs was associated with a significantly lower PFS (*p* = 0.0071) and poorer OS (*p* = 0.042). Patients with >3 FLs had a median PFS of 4.4 months vs. not reached for patients with ≤ 3 FLs and a 6 months-PFS of 35% vs. 94% (Figure 1). Among the 17 patients who experienced disease progression, 71% had >3 FLs. The presence of >3 FLs also impacted OS (*p* = 0.042) with a mean OS of 12 months in the group with >3 FLs vs. 18 months in the group with ≤3 FLs (median not reached in either group). A PET-derived bone SUV max superior to 11.5 (*n* = 8) was associated with both a lower PFS (*p* = 0.045) and OS (*p* = 0.045). The presence of EMD or BM with a Deauville score of ≥ 4 did not affect the patient outcomes significantly.

### 3.4. Prognostic Score before Anti-CD38 Immunotherapy

By multivariable analysis, only the ISS score (1 vs. 2 vs. 3) and > 3 FLs on PET/CT were found to be independent prognostic factors for PFS (*p* = 0.010, HR = 2.4 and *p* = 0.025, HR = 3.5 respectively). Concerning the OS outcome, only the ISS score reached significance (*p* = 0.032).

**Figure 2:**
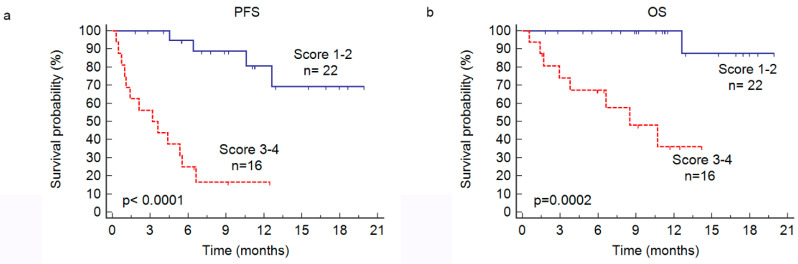
Kaplan–Meier estimates of progression-free survival (PFS, **a**) and overall survival (OS, **b**) in accordance with a prognostic score of 1–2 versus 3–4 points, which was a combination of the initial ISS score (1,2 or 3 points) with the presence of more than three focal lesions detected on PET (>3 FL: 1 point) or less than/equal to three FLs (≤3 FLs, 0 points).

We built a prognostic score before anti-CD38 therapy in our present RRMM cohort that combined the initial ISS (1, 2, or 3 points) with the presence of >3 FLs on FDG PET/CT at relapse (1 point). This enabled us to individualize two risk groups for the PFS (*p* < 0.0001) and OS (*p* = 0.0002) (Figure 2). The group with a score of 3–4 (*n* = 16) had a dismal outcome with a median PFS and OS of 3.1 months and 8.5 months, respectively vs. not reached for patients in the favorable group with a score of 1–2. This high-risk group included 42% of the study population but only 76% of the PFS events and 89% of the OS events.

## 4. Discussion

Daratumumab and Isatuximab were the first immunotherapies to have proved efficacy as a monotherapy in RRMM patients [9]. Nowadays, these agents are largely used in association with chemotherapy in MM patients after a first relapse. However, approximately 60% of these patients do not achieve a very good partial response and with a median time to progression of 12 months, the prognosis after relapse on anti-CD38 immunotherapy is poor [1]. There is notable inter-patient heterogeneity and additional prognostic markers are needed to determine which patients are at risk of an unfavorable outcome so that other therapeutic options can be considered as they become available.

This present study is the first to our knowledge to evaluate the prognostic impact of PET/CT parameters before the use of anti-CD38 monoclonal antibody treatment in RRMM patients. Our findings demonstrate that the presence of more than 3 bone focal lesions detected on a relapse PET may strongly influence patient outcomes in RRMM. Combined with the initial ISS, the assessment of FLs can be used to discern an ultra-risk RRMM population with a median PFS and OS of 3.1 months and 8.5 months respectively vs. not reached for the other patients.

18F-FDG PET/CT is considered a valuable tool for the visualization of disease activity in both newly diagnosed (ND) and RRMM patients [3,11], by providing a whole-body evaluation at once. It is also currently the IMWG recommended imaging technique for evaluating the response to therapy in MM. Indeed, several studies have demonstrated the value of PET/CT positivity as an indication of a poor response to treatment during the monitoring of MM patients [16,17,18,19,20,21]. Of note, in particular, FDG PET/CT, whether performed post-induction or post-ASCT, has been shown to have a higher prognostic value than MRI for the treatment response evaluation of newly diagnosed MM patients [18,22], even when compared with whole-body diffuse weighted MRI [20]. In addition, baseline FDG PET/CT parameters, such as the number of Fls and the presence of FDG PET/CT positive EMD, have been proven to be reliable outcome predictors in ND MM patients [16,22,23].

We demonstrate herein in an MM relapse setting that FDG PET/CT also has a strong prognostic value. In particular, our findings indicate that the presence of >3 FLs appears to be associated with a dismal outcome, with a median PFS of 4.4 months vs. not reached for patients with ≤3 FLs. In our present study cohort, about 75% of the patients who experienced a PFS event had >3 FLs. Bartel et al. [22] previously evaluate 239 newly diagnosed MM patients subsequently treated with the Total Therapy Three regimen and also found that the only imaging test significantly associated with an adverse prognosis for both overall survival (OS) and event-free survival (EFS) was FDG PET/CT with a number of FLs greater than three. The prognostic relevance of at least 3 FLs has also been described in a prior prospective series of newly diagnosed MM cases who received thalidomide-dexamethasone (TD) associated with double ASCT [16]. Further studies of MM have shown that at relapse, the presence of six FLs [24] or 10 [25] was an independent negative prognostic factor in terms of both the PFS and OS outcomes. In the context of daratumumab therapy, the CASSIOPET study, the PET/CT companion study of the CASSIOPEIA trial, showed that more patients reached PET/CT negativity in the Daratumumab-VTD (Bortezomib, Thalidomide, Dexamethasone) arm than in the VTD arm [26]. However, Daratumumab does not seem to be able to counteract the poor prognosis of the extramedullary disease [27]. Overall, to our knowledge, few studies have looked at the correlation between PET/CT and the response to anti-CD38 treatment.

Our study shows the importance of bone evaluation before initiation of treatment in the relapse setting. Cavo et al. [3] showed that FDG avidity provides an earlier evaluation of response to therapy and that FDG PET/CT at diagnosis is a predictive prognostic factor in patients with MM. Based on our results, it would be interesting to evaluate the impact of early PET/CT in the context of immunotherapy at relapse. Interestingly, our PET/CT results suggest that the tumor mass of myeloma at relapse appears similar to the tumor mass at diagnosis. Systematic bone evaluation could help to detect relapse sooner such as shown in solid cancers and metastases [28]. This should be correlated with biological evaluation.

Prognosis markers in MM have to date relies on the tumor burden and tumor biology. The ISS score (albumin and B2microglobuline) is a simple calculation that reflects MM infiltration and is largely used at diagnosis and in a relapsed setting [13]. Recently, other markers have been used such as the free light chain ratio or bone marrow infiltration. In our current study, the number of FLs was found to correlate with the B2microglobulin serum level confirming that they are two tumor burden surrogates. The presence of >3 FLs was also associated with a higher dFLC, even if this did not reach statistical significance. In line with these data, Bartel et al. reported previously that FDG PET/CT data showed the best correlation with biological markers such as B2microglobuline or the ISS, as compared with MRI or a whole body CT-scan [22]. Our current MM patients with >3 FLs have a higher ISS score, but in multivariable analysis, the PET/CT criteria and the ISS remained two independent prognostic factors for the PFS outcome. We thus propose a prognostic index combining the initial ISS (1, 2, 3 points) with the presence of >3 FLs on relapse PET/CT (1 point). This index could then be used to stratify high-risk MM patients which included only 42% of the current study population but accounted for 76% of PFS events and 89% of the OS events.

The detection of cytogenetic abnormalities at baseline (t(4,14); t(14–16), del17p) is a well-known method of identifying MM patients at high risk of relapse, even in a case with a low tumor burden and immunochemical response [29]. In our series, however, 24% of the cases had these high-risk cytogenetic abnormalities this was not found to be significantly associated with the PFS and OS outcomes. This may have been due to the small number of patients in our cohort and a lower resulting statistical power. Another explanation may have been prognostic improvements among these cases due to anti-CD38 therapy. By contrast to the report of Zhou et al., we did not find a high SUVmax in patients harboring del(17p) compared with those without this abnormality [30]. At baseline, a high bone SUVmax has been suggested to be a prognostic indicator [31]. In our present analysis, a high SUVmax impacted the PFS and OS outcomes but was far less significant than the presence of >3 FLs.

At the time of an MM relapse, the prognosis depends not only on disease biology but also on the patient’s characteristics and reaction to the disease and an ability to tolerate intensive therapy. Indeed, symptomatic patients have a poorer prognosis than those with only an immunochemical relapse [6]. Anemia is a well-known prognostic factor in patients with cancer as it reflects inflammation, malnutrition, and poor general health status [7]. In an MM setting, low hemoglobin is a prognostic factor for survival [8]. In our present cohort, anemia at relapse was the single biological parameter that correlated both with poorer PFS (*p* = 0.030) and OS (*p* = 0.0096). We confirmed that patients showing symptomatic relapse characterized by focal bone uptake on PET/CT and also with anemia have both a poorer PFS and OS.

Several authors have demonstrated that the presence of circulating plasma cells in peripheral blood is a prognostic biomarker that could be used for risk stratification in MM [32,33,34,35,36]. We, therefore, wanted to analyze this parameter in our series, in the context of anti-CD38 therapy. However, the analysis could be done in only 15 patients and was positive in 5 patients, which unfortunately limits the analysis of our data.

This pilot study had other several limitations of note. First, this was a single-center retrospective study. Due to the retrospective nature of the data collection, some information regarding cytogenetic abnormalities was missing and this may explain why high-risk cytogenetics were not found to be associated with survival outcomes. In addition, the size of this cohort is small, which limits the statistical power. Our present findings should thus be interpreted with caution given the small sample size and need to be confirmed in larger multicenter studies. Nevertheless, these results have given important new insight into the prognostic value of PET/CT in RRMM patients treated with anti-CD38 immunotherapy and have provided a solid rationale for further investigations.

## 5. Conclusions

The present study shows that FDG PET/CT can be used for prognostic purposes in patients with RRMM treated with anti-CD38 immunotherapy. At relapse, the presence of more than 3 bone FLs detected on PET/CT is predictive of a poorer PFS and OS. Combined with the initial ISS, this simple PET criterion could improve the risk stratification of RRMM patients and thus enable the earlier and better identification of ultra-risk patients.

## Figures and Tables

**Table 1:** Patients characteristics (*n* = 38), for the whole cohort and according to the presence of ≤3 FLs or >3 FLs on PET/CT.

Characteristics	All Patients *n* = 38	PET ≤ 3 FLs *n* = 19	PET > 3 FLs *n* = 19	*p*
*Clinical characteristics at relapse*				
Age, median (range)	73 (58–87)	75 (62–87)	69 (58–87)	0.58
Age > 75 years, *n* (%)	17 (45)	10 (53)	7 (37)	0.51
Sex ratio male/female	1 (19/19)	0.7 (8/11)	1.37 (11/8)	0.52
*Biological parameters at relapse*				
dFLC mg/L, median (IQR) *	180 (18–946)	85 (10–437)	206 (103–1142)	0.08
Abnormal FLC ratio, *n* (%) *	25 (66)	11	14	0.29
High FLC ratio (>120), *n* (%)	6 (16)	1	5	0.18
Creatinine clearance < 60 mL/min	14 (37)	7	7	1
Hemoglobin < 10 g/dL, *n* (%)	11 (29)	3	8	0.15
β2microglobulin (mg/L) *	3.67 (1.3–14.2)	2.75 (1.3–9.6)	4.13 (1.8–19)	0.015
Elevated LDH, *n* (%)	12 (32)	6	6	1
*Initial myeloma characteristics*				
Isotype				
IgG, *n* (%)	21 (55)	10	11	0.95
IgA, *n* (%)	8 (21)	4	4	
Light chain only (kappa), *n* (%)	9 (24)	5	4	
ISS stage				
ISS 1, *n* (%)	16 (42)	12	4	0.028
ISS 2, *n* (%)	14 (37)	5	9	
ISS 3, *n* (%)	8 (21)	2	6	
Cytogenetics				
High-risk cytogenetics, *n* (%)	8 (21)	2	6	0.24
No High-risk cytogenetics, *n* (%)	25 (66)	13	12	
Missing	5 (13)	4	1	
*Therapy*				
Line of therapy before antiCD38	3 (2–10)	3 (2–4)	3 (2–10)	0.48
>2 previous lines of therapy, *n* (%)	20 (53)	10	10	1
ASCT transplantation, *n* (%)	18 (47%)	8	10	0.74
Type of anti-CD38				
Daratumumab, *n* (%)	32 (84)	18	14	0.18
Isatuximab, *n* (%)	6 (16)	1	5	
Associated treatment				
Corticosteroids only, *n* (%)	7 (18)	2	5	0.40
ImiD, *n* (%)	19 (50)	10	9	1
Proteasome inhibitor, *n* (%)	10 (26)	6	4	0.71
Other, *n* (%) ^1^	4 (11)	1	3	0.60

^1^ Chemotherapy (2), Azacitidine for concomitant MDS (1), Trametinib (1). ASCT: Autologous stem cell transplantation FLC: serum immunoglobulin-free light chain. * missing data.

**Table 2:** PET/CT parameters.

PET/CT Parameters	Patients (*n* = 38)
Presence of FLs, DS score	26
2	0
3	3
4–5	23
Presence of >3 FLs	19
Presence of EMD	7
Median FL SUVmax (IQR)	7.8 (5.1–13.8)
Median EMD SUVmax (IQR)	12.9 (6.8–17.7)
Median liver SUVmax (IQR) *	3.2 (2.7–3.5)
Presence of BM uptake, DS score	
<4	31
≥4	7

* exclusion of one patient with liver myeloma infiltration.

**Table 3:** Cox regression analyses of the clinical, biological, and FDG PET/CT parameters associated with overall and progression-free survival.

Parameters	PFS		OS	
	HR (95%CI)	*p*	HR (95%CI)	*p*
Initial MM characteristics				
Initial ISS score (1 vs. 2–3)	4.4 (1.3–15.3)	0.019	7.0 (0.9–55.1)	0.066
Initial ISS score (1–2 vs. 3)	4.0 (1.5–10.7)	0.0065	6.6 (1.6–27.8)	0.010
Initial ISS score (1 vs. 2 vs. 3)	2.7 (1.4–5.1)	0.0026	3.8 (1.4–10.6)	0.0098
High-risk cytogenetics	1.2 (0.4–3.7)	0.79	1.0 (0.2–5.2)	0.95
Previous treatments				
>2 previous line	1.1 (0.4–2.9)	0.85	1.6 (0.4–6.3)	0.51
Previous ASCT	0.6 (0.2–1.8)	0.41	0.4 (0.7–1.7)	0.21
Biological parameters before anti- CD38 therapy				
β2microglobulin ≥ 3.5 mg/	2.8 (0.9–8.5)	0.073	6.0 (0.7–48)	0.089
β2microglobulin > 5.5 mg/L	1.7 (0.6–4.8)	0.31	3.1 (0.8–11.7)	0.098
dFLC	1.0 (0.9–1.0)	0.19	1 (1.0–1.0)	0.013
Abnormal FLC ratio	2.5 (0.7–8.8)	0.15	-	0.95
Albumin ≤ 3.5 g/dL	2.6 (0.9–7.7)	0.082	1.3 (0.3–6.5)	0.72
Hb ≤ 10 g/dL	2.9 (1.1–7.5)	0.030	6.3 (1.6–25)	0.0096
LDH upper than normal	1.1 (0.4–3.1)	0.83	2.1 (0.6–7.9)	0.26
PET parameters before anti-CD38 therapy				
Presence of at least one FL	1.4 (0.5–4.1)	0.48	1.8 (0.3–8.5)	0.47
Presence of >3 FLs	4.3 (1.5–12.5)	0.0071	5.2 (1.1–25.4)	0.042
Presence of EMD	2.4 (0.8–7.8)	0.11	2.5 (0.4–14.5)	0.17
Lesion SUVmax > 11.5	2.8 (1.0–7.7)	0.045	3.9 (1.0–14.5)	0.045
Bone marrow ≥ DS 4	1.4 (0.5–4.4)	0.52	2.2 (0.5–8.9)	0.26

dFLC = difference between involved and uninvolved light chains.

## Data Availability

The data presented in this study are available on request from the corresponding author.
